# The *Plasmodium falciparum *merozoite surface protein-1 19 KD antibody response in the Peruvian Amazon predominantly targets the non-allele specific, shared sites of this antigen

**DOI:** 10.1186/1475-2875-9-3

**Published:** 2010-01-04

**Authors:** Patrick L Sutton, Eva H Clark, Claudia Silva, OraLee H Branch

**Affiliations:** 1 Department of Medical Parasitology, New York University, New York, New York, USA; 2 Department of Microbiology, University of Alabama at Birmingham, Alabama, Birmingham, USA; 3 Laboratorio de Investigación de Productos Naturales Antiparasitarios de la Amazonía, Universidad Nacional de la Amazonía Peruana, Iquitos, Perú

## Abstract

**Background:**

*Plasmodium falciparum *re-emerged in Iquitos, Peru in 1994 and is now hypoendemic (< 0.5 infections/person/year). Purportedly non-immune individuals with discrete (non-overlapping) *P. falciparum *infections can be followed using this population dynamic. Previous work demonstrated a strong association between this population's antibody response to *Pf*MSP1-19KD and protection against febrile illness and parasitaemia. Therefore, some selection for *Pf*MSP1-19KD allelic diversity would be expected if the protection is to allele-specific sites of *Pf*MSP1-19KD. Here, the potential for allele-specific polymorphisms in this population is investigated, and the allele-specificity of antibody responses to *Pf*MSP1-19KD are determined.

**Methods:**

The 42KD region in *Pf*MSP1 was genotyped from 160 individual infections collected between 2003 and 2007. Additionally, the polymorphic block 2 region of *Pfmsp1 *(*Pfmsp1*-B2) was genotyped in 781 infection-months to provide a baseline for population-level diversity. To test whether *Pf*MSP1-19KD genetic diversity had any impact on antibody responses, ELISAs testing IgG antibody response were performed on individuals using all four allele-types of *Pf*MSP1-19KD. An antibody depletion ELISA was used to test the ability of antibodies to cross-react between allele-types.

**Results:**

Despite increased diversity in *Pfmsp1*-B2, limited diversity within *Pfmsp1*-42KD was observed. All 160 infections genotyped were Mad20-like at the *Pfmsp1*-33KD locus. In the *Pfmsp1*-19KD locus, 159 (99.4%) were the Q-KSNG-F haplotype and 1 (0.6%) was the E-KSNG-L haplotype. Antibody responses in 105 individuals showed that Q-KNG and Q-TSR alleles generated the strongest immune responses, while Q-KNG and E-KNG responses were more concordant with each other than with those from Q-TSR and E-TSR, and vice versa. The immuno-depletion ELISAs showed all samples responded to the antigenic sites shared amongst all allelic forms of *Pf*MSP1-19KD.

**Conclusions:**

A non-allele specific antibody response in *Pf*MSP1-19KD may explain why other allelic forms have not been maintained or evolved in this population. This has important implications for the use of *Pf*MSP1-19KD as a vaccine candidate. It is possible that Peruvians have increased antibody responses to the shared sites of *Pf*MSP1-19KD, either due to exposure/parasite characteristics or due to a human-genetic predisposition. Alternatively, these allelic polymorphisms are not immune-specific even in other geographic regions, implying these polymorphisms may be less important in immune evasion that previous studies suggest.

## Background

If a population of parasites is able to evolve many different allelic forms of its antigenic proteins while still maintaining the biological function of each protein, it will increase the ability of the parasite population as a whole to evade immune responses. As a result, genetic diversity in antigenic encoding genes can indicate evidence of protective immune responses. However, diversity can also occur through random mutation. If the mutations are not lethal, they might be fixed in the population by random genetic drift. Such a process is very likely in *Plasmodium falciparum*, where populations undergo frequent constrictions (i.e. a genetic bottleneck, by drug selection pressure) and subsequent clonal expansion/replacement that is not due to selection. One powerful way to distinguish genes under immune selection pressure from those that are varying as a result of genetic drift is to combine genotyping data with antibody data, to establish whether the antigen in question generates an allele-specific immune response - that is, an immune response that differentially targets and kills parasites having different allelic forms.

It is suggested that the most promising malaria vaccine candidates are those which are developed against immunogenic regions of proteins that have been evolutionarily conserved due to functionality constraints, and those in which diversity is limited enough that is not likely to compromize overall vaccine efficacy. A leading vaccine candidate is the C-terminal 19KD portion of the *Plasmodium falciparum *merozoite surface protein-1 (*Pf*MSP1). *Pf*MSP1 has a primary structure (195KD) [1] that can be divided into 17 blocks based on the conservation and variability of the amino acid sequence [2-4]. *Pf*MSP1-33KD (encoded by block 16) is dimorphic, existing in two major allelic forms: K1-like and Mad20-like. Both of these alleles are genetically conserved with few synonymous and non-synonymous amino acid substitutions within each allele class [2,4]. *Pf*MSP1-19KD (encoded by block 17) is also relatively conserved, with only a few single nucleotide polymorphisms (SNPs) identified in its two epidermal growth factor-like (EGF-like) domains. These *Pf*MSP1-19KD EGF-like domains appear to be functionally critical for erythrocyte invasion and elicit anti-parasite immune responses [4-6]. The amino acid polymorphisms in *Pf*MSP1-19KD appear to have evolved in order to evade the human immune response capable of blocking the parasite from invading red blood cells [4,7-10] and are located in six known locations: 1644 (E/Q), 1691 (K/T), 1699 (N/S), 1700 (N/S), 1701 (R/G) and 1716 (F/L) [4,11,12]. The dimorphic nature of the amino acid polymorphism and the resulting major forms of E-KNG, E-TSR, Q-KNG and Q-TSR suggest that some of these alleles emerged by recombination, although this might also be due to functional constraints inhibiting multiple changes of certain sites in one evolutionary leap.

To determine if the four *Pf*MSP1-19KD main allelic forms result in naturally acquired allele-specific immunity, studies have compared antibody responses among individuals naturally exposed to *P. falciparum *malaria. A study by Shi *et al *[13] compared antibody responses against the four major allele-types of *Pf*MSP1-19KD in a population of individuals highly exposed to all allelic forms [13]. Not surprisingly, individuals could respond to various alleles. However, it was noted that the anti-E-KNG and anti-Q-KNG responses correlated more closely with each other, and similarly the anti-E-TSR and anti-Q-TSR responses correlated more closely, suggesting that the -KNG and -TSR epitopes are targets of allele-specific immunity [13]. In a more recent study, Mamillapalli *et al *[14] developed an assay to test allele-specific cross-reactivity/specificity [14]. By conducting an immuno-depletion assay on sera from three acutely infected individuals (who had been previously infected with many different allelic types), they observed antibody responses to both allele-specific and shared sites [14].

In order to advance vaccine candidate antigens such as *Pf*MSP1-19KD along the development pipeline, the range of antigenic diversity in the targeted population must be known, and also the likelihood that the antigen generates antibody responses to shared sites that might be associated with protection. The goal of the current study was to determine if there are different *Pf*MSP1-19KD allelic forms circulating at the study site in Iquitos, Peru, and whether *P. falciparum *infected individuals at the study site develop antibody responses to shared (conserved) sites, or whether the responses are allele-specific to the four main family alleles: E-KNG, Q-KNG, E-TSR, and Q-TSR.

Since 2003, a longitudinal active case detection cohort study, (Malaria Immunology and Genetics in the Amazon, MIGIA) has been conducted in one of the highest malaria transmission communities near Iquitos, Peru, called Zungarococha, where *P. falciparum *malaria re-emerged in 1994 after more than 30 years of convalescence. Since the 1994-1998 epidemic there has been sustained low transmission in this region (< 0.5 *P. falciparum *infections/person/year in Zungarococha since 2003) [15,16]. From infections occurring between 2003 and 2007, *Pfmsp1*-42KD (*Pfmsp1*-33KD + *Pfmsp1*-19KD) was PCR-genotyped to determine family-type, and then *Pfmsp1*-19KD was sequenced to determine the genetic diversity in this region. Additionally, the population-level diversity was further characterized by genotyping the polymorphic *Pfmsp1*-Block 2 (*Pfmsp1*-B2) as a baseline for the potential population-level diversity. *Pf*MSP1-19KD antibody responses at the study site were then measured to the four major *Pf*MSP1-19KD allele-types and antibody depletion ELISA experiments were performed to determine the degree of cross-reactivity in the antibody responses to each *Pf*MSP1-19KD allele-type. The results have implications for the development of *Pf*MSP1 based vaccines.

## Methods

### Study design

Blood samples were collected in Zungarococha (N = 1907) during the malaria transmission season (January-July) from 2003 to 2007. Details on the study site and design are described by Branch *et al *[16]. In brief, active case detection included a beginning and ending malaria season community-wide cross-sectional survey and also a selection of approximately 200 individuals each month during the malaria season for weekly visits for one month. Additionally, there was passive case detection executed in the health center. This study had ethical approval from U.S.A and Peruvian ethical review boards, and all participants provided informed consent and/or assent, and these approvals continue with annual review.

In the active case detection, blood slides from individuals who had a body temperature of ≥38.3°C, reported having a high fever within two days, or had a haematocrit < 30% pcv, had their blood slides read by expert microscopists within one day and were treated within one day if positive. If an individual was asymptomatic, there could be six days before reading the microscopy slide. At the next scheduled visit (one week later) another blood sample was collected and any individual who was found with malaria parasites on the week before visit had their blood slide read immediately. Therefore, asymptomatic individuals have more than one blood slide collected during a given infection before treatment. Treatment regimens are reported in Branch *et al *[16].

### Sample selection for genotypification of *Pfmsp1*-33KD and *Pfmsp1*-19KD

*Plasmodium falciparum *infections were detected by microscopy or by polymerase chain reaction (PCR) [16,17]. Sample size was estimated by comparing a known proportion to an anticipated proportion, accounting for the detection of rare alleles occurring in this population at a rate of at least one percent with a power of 80% [18]. To adequately represent the population in this cohort, gender, age, community, and year of infection were considered when selecting samples.

### PCR-genotyping of *Pfmsp1*-33KD and *Pfmsp1*-19KD

**Figure 1:**
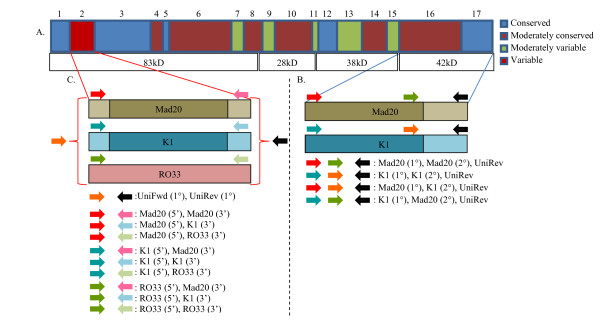
**A-C - Genotyping methods for *Pfmsp1*-42KD and *Pfmsp1*-B2**. A) Schema of *Pfmsp1*, indicating contrasting regions of conservation and variability. B) Highlights the *Pfmsp1*-42KD region of *Pfmsp1*. The polymorphic nature of *Pfmsp1*-33KD is indicated by the darker shades at the C-terminal end of the Mad20 allele (tan) and K1 allele (blue), while the lighter shades are used to indicate increased conservation. Colored arrows, defined in the figure, are used to illustrate the position of the primers used in PCR amplification. The colored arrows, with the same definition as found in (A), are used to illustrate the combination of primers (Mad20 or K1) used when testing for permutations that may have been observed due to interfamily sexual recombination. C) Highlights the three allelic families detected as a result of using the Roberts et al [22] method. Stemming from the polymorphic *Pfmsp1*B2 region, illustrated as the red block, are those three allelic families: Mad20 (tan), K1 (blue), RO33 (pink). Darker shades at the center of Mad20 and K1 are used to indicate increased genetic variation due to it the presence of a variable repeat-length region. Colored arrows, defined in the figure, are used to illustrate the position of the primers used in PCR amplification. The colored arrows, with the same definition as found in (A), are used to illustrate the combination of primers (5' or 3') used when testing for permutations that may have been observed due to interfamily sexual recombination.

All samples selected for this study underwent a semi-nested-PCR reaction. Due to the dimorphic nature of *Pfmsp1*-42KD, family-specific primers for the Mad20 and K1 allelic families were designed for both primary and secondary PCRs. To test whether recombinatory events were occurring within these regions, all permutations of these primers were tested on all samples. Primers were designed from previously established sequences [4]. The primary PCR amplifies an approximate 1200 bp region at the C-terminal end of *Pfmsp1*, including both *Pfmsp1*-33KD and *Pfmsp1*-19KD. Two primary PCRs were performed using family-specific primers: Mad20 forward 5'-GCAATATCTGTCACAATGG (1358) or K1 forward 5'-GCAGTAACTCCTTCCGTAATTG (1329) in combination with a universal reverse 5'-TTAGAGGAACTGCAGAAAATACCA (1729). Four secondary PCRs were performed using amplified/non-amplified product form the primary PCR, accounting for all possible permutations of these primers. African clones positive for each individual allele-type were used as positive controls. This PCR amplifies *Pfmsp1*-19KD and an N-terminal flanking region (approximately 450 bp): Mad20 middle forward 5'-CCATAACGACTTCGAAGC (1580) or K1 middle forward 5'-CGTTGGAATTGCTGATTTATCAACAG (1585), and the universal reverse (Figure 1, A and 1B).

Both primary and secondary PCRs were initially performed in a 25 μL total reaction volume. If there was amplification in the secondary PCR, the secondary PCR was performed again at an increased total reaction volume of 100 μL for sequencing. Mastermixes for primary and secondary PCRs were comprised of 5' and 3' primers [0.8 mM] (Integrated DNA Technologies, Inc, Coralville, IA, USA), deoxynucleoside triphosphate (dNTP) mixture [0.4 mM] (Invitrogen™, Carlsbad, CA, USA), MgCl_2 _[1 mM] (Promega, Madison, WI, USA), 5× PCR buffer, 1 U of *Taq *polymerase (Promega Go *Taq *Flexi, Madison, WI, USA), molecular grade water, and blood extracted DNA (genomic DNA adjusted to 20-40 ng/μL) or externally amplified (PCR amplicon) DNA. All PCRs were performed in an Eppendorf Mastercycler^® ^ep (Westbury, NY, USA). Mastermixes were adjusted accordingly for increases in total reaction volume.

Cycling conditions for the primary reaction were as follows: denature at 94°C for 1-min, 1 cycle; denature at 94°C for 45-sec, anneal at 55°C for 45-sec, extension time of 1.5-min at 60°C, 40 cycles (35 cycles for secondary PCR); and a final extension cycle of 60°C for 5-min. Products from each primary and secondary PCR were individually visualized by UV illumination after gel electrophoresis using a 1.5% concentration of Agarose, Genetic Technology Grade (MP Biochemicals, LLC, Solon, OH, USA). Each allele was characterized by family allele-type based upon visualization of bands after amplification with family-specific primers. A 100 bp DNA ladder (Invitrogen™, Carlsbad, CA, USA) was used to determine product size.

### Sequencing of *Pfmsp1*-19KD

Products from secondary PCRs performed in 100 μL total reaction volume were individually visualized by UV illumination after gel electrophoresis using a 1.5% concentration of UltraPure™ Agarose 1000 (Invitrogen™, Carlsbad, CA, USA). PCR products were isolated and then purified using a Gel Extraction Kit (Qiagen^®^, Valencia, CA, USA). Purified PCR products at a final concentration of 10 ng/μL were sequenced. The 5' and 3' primers [2.0 μM] for each allelic family were used to sequence their respective alleles. All sequencing was performed on an ABI 3730 Genetic Analyzer (Applied Biosystems, Foster, CA, USA) by the University of Alabama at Birmingham, Howell and Elizabeth Heflin Center for Human Genetics. Sequences were preliminarily viewed using Chromas 2.33 (^©^2003-2008 Technelysium Pty Ltd), but aligned using ClustalX2 [19].

### PCR-genotyping of *Pfmsp*1-B2

*Pfmsp1*-B2 genotyping was attempted on all available samples that were microscopy and/or PCR positive for *P. falciparum *between 2003 and 2007 (781 *P. falciparum *infection-months). Given the active case detection study design (with frequent sampling during follow-up) it was possible that an individual could be sampled multiple times over the course of weeks. Infections were defined as one or more malaria-positive sample point(s) during the follow-up. All sampled time points within one month before and after the infection were considered. The sampling could begin before the initial malaria positive sample or after, with an overall sampling time frame of ≤30 days (four weeks of sampling follow-up planned). For this reason, infections are called "infection-months," and this is the denominator used when considering the number of infections followed and the frequency of the different parasite alleles detected [20]. The appendence of 2005-2007 genotyping data to previously reported years 2003-2004 [20] allows for the establishment of a continued baseline of the potential population-level diversity. Previously reported genotyping methods were used for main allelic family analysis [20-23]. Additionally, all permutations of primers were used to detect any allelic families that are the result of recombination (Figure 1, A and 1C). PCR products for the K1, Mad20, and RO33 allelic families were observed; no Mad20-RO33 recombinant alleles were observed [20,21].

### Measurement of IgG

ELISA for total IgG was performed as described previously [24,25]. Plates (Nunc-Immuno™ Polystyrene Plates, A Part of ThermoFisher Scientific, Rochester, NY, USA) were coated with 50 μL/well of recombinant *Pf*MSP1-19KD (E-KNG, Q-KNG, E-TSR, or Q-TSR) at a concentration of 0.25 ng/μL. Recombinant E-KNG, E-TSR, and Q-KNG *Pf*MSP1-19KD antigens were obtained from the MR4 division of ATCC (Manassas, VA). Recombinant Q-TSR was provided by David Kaslow. The plates were then blocked with BBS and 1% Bovine Serum Albumin (BSA). Sera samples were diluted 1:100 in Sera Dilution Buffer (1.5% nonfat milk in AB washing solution [0.15 M Na_2_HPO_4_, 0.15 M NaH_2_PO_4_, NaCl, 0.05% Tween20, and 0.05% BSA]) and added to plates coated with *Pf*MSP1-19KD antigens. After incubating the plates for two hours and then washing them with AB Wash, 50 μL of secondary antibody (horseradish peroxidase-conjugated goat-anti-human-IgG (Chemicon, Millipore™ Billerica, MA, USA) was added to each well at a concentration of 1:4000. After a second two-hour incubation period, the plates were again washed with AB Wash and 50 μL of 3,3', 5,5'-tetramethylbenzidine (KPL, Inc., Gaithersburg, MD, USA) was added to each well. The reaction was stopped after approximately six minutes using 25 μL of 0.25 M HCl per well. The plates were read at 450 nm (A_450_) with an ELISA plate-reader (Bio-Rad, Heracles, CA, USA).

Six serum samples from healthy non-exposed Peruvians were used as negative controls. The positive controls included a "positive pool" made-up of 5 different *P. falciparum*-infected individuals. A negative cut-off value (the average of the negative control samples plus two times the standard deviation of the negative controls) was calculated for each experiment day to determine the difference between negative and positive optical density (OD) thresholds for each antigen.

### Statistical analysis of *Pf*MSP1-19KD ELISA data

The statistical study of the antibody response data for the *Pf*MSP1-19KD allele-types was completed in two analyses. The first compared paired, continuous OD values (N = 105), while the second compared the frequencies of categorical classifications (positive versus negative) of each value. In both analyses, "positive" describes any result greater than the negative cut-off value and "negative" describes any value less than or equal to the negative cutoff value. All statistical analyses were performed using GraphPad Prism (version 4.00 for Windows, GraphPad Software, San Diego, California, USA).

### Antibody depletion ELISAs

**Figure 2:**
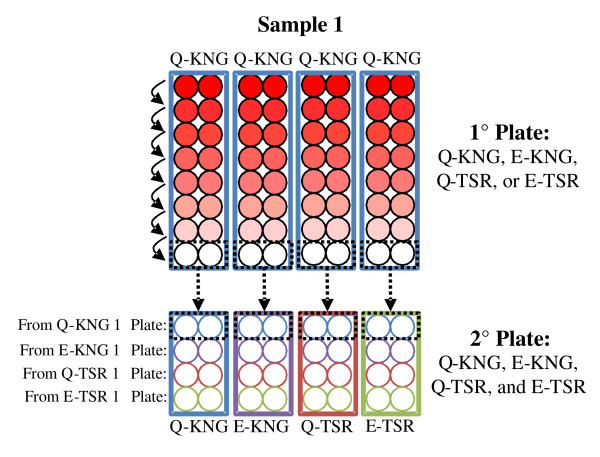
**Experimental design for Antibody depletion ELISA**. A representative sample is shown. Patients' sera were plated in duplicate and in 4 replicates in the first row on each type of "primary" plate (E-KNG, Q-KNG, E-TSR, and Q-TSR). The sera were then transferred down the plate seven times, incubating for half an hour before each transfer. After incubating the patient sera in the last row of the primary plate, it was transferred to a secondary plate of each allele.

To evaluate the cross-reactivity of antibodies against each *Pf*MSP1-19KD allele in sera samples from 18 different individuals, an ELISA was performed after antibody depletion with each of the allelic forms. This method was similar to the immunoassays used by Mamillapalli *et al *[14], where the assay was called "Antibody depletion ELISAs." After diluting the patient sera at 1:100 in AB Wash + 1.5% milk, the sera solution was placed in the first row of a 96-well primary plate (primary plates were coated with 50 ng of antigen per well in the first row of the plate and 100 ng of antigen per well in the seven consecutive rows). Patient sera were plated separately on E-KNG, Q-KNG, E-TSR, and Q-TSR primary plates. After incubating the sera for 30 min, it was then transferred to the next row of wells, where it was again incubated for 30 min. The wells of the primary plate were washed once with 50 μL of AB wash after each transfer, and the residual wash buffer was then placed with the sera to recover as much antibody as possible from the primary plates. Seven such transfers were performed. After the last transfer, the sera was transferred to secondary ELISA plates--one secondary plate for each of the four allele-types coated at 50 ng of antigen per well in all wells (Figure 2)--where it was incubated for one hour, and then the wells of both the primary and secondary plates were washed 4 times with AB wash and the ELISA was completed as described above in "Measurement of IgG."

## Results

### Genetic diversity of *Pf*MSP1-33KD and *Pf*MSP1-19KD

From 781 *P. falciparum *infection-months with DNA available for testing, DNA samples were selected representing 187 infections (participants). Sample selection was guided by gender, age, community, and year of infection (Table 1). The genotyping results were clear for all 187 samples, but reliable sequences were only achieved on 160 of the 187 samples.

**Table 1:** Distribution of population variables used in sample selection for *Pfmsp1*-19KD genotyping

Variable		N	Distribution
Gender	Male	80	0.50
	Female	80	0.50

Age	≤14-years	60	0.38
	≥15-years	100	0.63

Collection Year	2003	29	0.18
	2004	48	0.30
	2005	41	0.26
	2006	42	0.26

Community	Llanchama	25	0.16
	Ninarumi	54	0.34
	Puerto Almendra	36	0.23
	Zungarococha	29	0.18
	Other	16	0.10

There was no allelic variation observed within *Pf*MSP1-33KD, as all 160 participant samples were identified as the Mad20 allele-type in this region. Primer permutations were used in the secondary PCR to determine if recombinatory events had occurred in recent evolutionary history between family allele-types in this region. The semi-nested secondary PCR did not suggest recombination throughout *Pf*MSP1-33KD. When *Pf*MSP1-19KD was sequenced, 159 (99.4%) samples were identified as the Q-KSNG-F variant, while only one sample (0.6%) was identified as the E-KSNG-L variant. The E-KSNG-L variant was detected in year-2003 and not in the four subsequent years of this study (Table 2).

**Table 2:** *Pfmsp1*-42KD PCR genotyping and sequencing results

*Pfmsp1*-33KD	N	Frequency	*Pfmsp1*-19KD SNP and Position						GenBank No.
			**1644**	**1691**	**1699**	**1700**	**1701**	**1716**	

Mad20	159	99.4%	**Q**	K	S	N	G	**F**	FJ959104
Mad20	1	0.6%	**E**	K	S	N	G	**L**	FJ959105

### Genetic diversity of *Pfmsp1*-block 2

There were 951 *Pfmsp1*-B2 alleles detected in the 781 infection-months in this study from 2003-2007. Of the 781 infection-months, 80.0% were single infections, while 20.0% were complex infections (defined by the detection of more than one *Pfmsp1*-B2 allele in a single sample or throughout the infection-month).

The K1, Mad20, and RO33 allelic families that have been detected globally in prior studies were detected in Zungarococha. Three K1 allele-types (170 bp, 195 bp, 220 bp), three Mad20 allele-types (200 bp, 210 bp, 230 bp), and one RO33 allele-type (140 bp) were observed. Of the 951 alleles detected, 499 (52.5%) were K1, 431 (45.3%) were Mad20, and 21 (2.2%) were RO33 (Table 3). Though the K1 and Mad20 allelic families appear to be equally distributed, when these infections were analysed over time there was a decrease in the detection of K1 allele-types (74.7% to 44.7% between 2003-2007), complemented by an increase in the detection Mad20 allele-types (20.2% to 55.3% between 2003-2007). The RO33 main allelic family was observed at a low frequency throughout all years and as part of a mixed infection in all instances but one. Individual allele-types for *Pfmsp1*-B2 allelic families can be found in Table 3.

**Table 3:** *Pfmsp1*-B2 main allelic family allele-type frequencies

Main Family	Allele-type (bp)	Frequency (N = 951)
K1	170	12.1%
	195	39.9%
	220	0.5%

Mad20	200	4.9%
	210	39.6%
	230	0.7%

RO33	140	2.2%

### Antibody responses to *Pf*MSP1-19KD IgG

Total IgG antibody responses to all four *Pf*MSP1-19KD allele-types were tested on 105 samples. Of the 105 individuals, 62 (59.1%) were positive for all alleles, and 27 (25.7%) were negative for all alleles. Considering the optical density (OD) readings, the mean values for Q-KNG and Q-TSR were the highest of the four alleles, (1.139 and 0.983, respectively), followed by EKNG (mean = 0.963) and ETSR (0.904) (Table 4).

**Table 4:** Descriptive Statistics for *Pf*MSP1-19KD antigens

	E-KNG	Q-KNG	Q-TSR	E-TSR
**Number of values**	105	105	105	105

**Minimum**	-0.435	0.051	0.029	-0.009
**25% Percentile**	0.308	0.465	0.200	0.164
**Median**	0.971	1.181	0.968	0.796
**75% Percentile**	1.585	1.772	1.695	1.625
**Maximum**	2.126	2.081	2.109	2.075

**Mean**	0.963	1.139	0.983	0.904
**Std. Deviation**	0.663	0.629	0.696	0.693
**Std. Error**	0.065	0.061	0.068	0.068

**Lower 95% CI of mean**	0.835	1.017	0.848	0.770
**Upper 95% CI of mean**	1.091	1.260	1.118	1.038

**Coeff. of variation**	68.84%	55.23%	70.84%	76.62%

However, a one-way analysis of variance (ANOVA, Friedman test) comparing values for all four *Pf*MSP1-19KD allele-types indicated that there was a significant difference between variances (P < 0.0001; Friedman statistic = 58.25) when compared together. Table 5 shows the between groups post-tests performed during this analysis, indicating that there were significant differences between Q-KNG and E-KNG, Q-KNG and Q-TSR, and Q-KNG versus E-TSR (all P < 0.0001), while there were no significant differences between any of the other allelic combinations.

**Table 5:** One-way ANOVA: E-KNG vs Q-KNG vs Q-TSR vs E-TSR

Dunn's Multiple Comparison Test	Summary
E-KNG vs Q-KNG	***
E-KNG vs Q-TSR	ns
E-KNG vs E-TSR	ns
Q-KNG vs Q-TSR	***
Q-KNG vs E-TSR	***
Q-TSR vs E-TSR	ns

### Allele-specific/shared site responses

**Figure 3:**
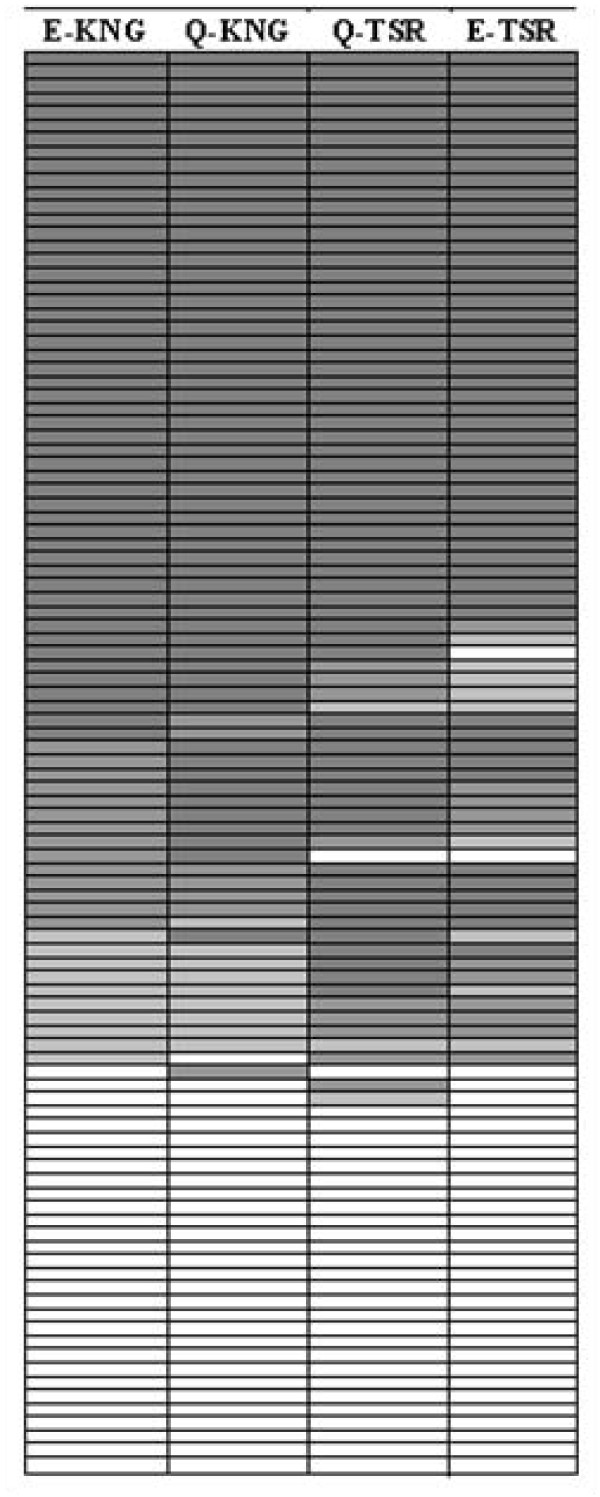
**Tabular comparison of concordance between the four *Pf *MSP1-19KD alleles**. N = 105, dark grey = high positive antibody response (values greater than 2 * negative cut-off for each allele), medium grey = medium positive response (values between 1.5 * the negative cut-off and 2 * the negative cut-off), light grey = low positive response (values between the negative cut-off and 1.5 * negative cut-off), and white = negative antibody response (values less than the negative cut-off). Each row represents a different code/date.

First, individuals' responses to each *Pf*MSP1-19KD allele-type were considered. Figure 3 shows positive (stratified into high positive [HP], medium positive [MP], and low positive [LP]) and negative categorical values for each allele in a pair-wise manner. E-KNG responses matched Q-KNG responses 85.7% of the time and E-TSR 77.1% of the time. However, the E-KNG responses matched Q-TSR responses only 73.3% of the time. Similarly, Q-KNG matched Q-TSR 77.1% and E-TSR 72.4% of the time. Q-TSR and E-TSR were consistently close in value 83.8% of the time.

In a paired correlation analysis comparing the continuous OD measurements to the four allele-types, E-TSR and Q-TSR were found to correlate at the highest rate (correlation coefficient = 0.938, see Table 6), although E-KNG and Q-KNG had a similarly high correlation coefficient (0.935). The lowest correlation coefficient was found to be between E-KNG and E-TSR, but it was still relatively high at 0.852. Correlations between all pairings were significant (P < 0.0001).

**Table 6:** Correlation analysis between the 4 *Pf*MSP1-19KD alleles

	E-KNG		Q-KNG		Q-TSR		E-TSR	
	**Correl. Coeff**	**P value**	**Correl. Coeff**	**P value**	**Correl. Coeff**	**P value**	**Correl. Coeff**	**P value**

**EKNG**	-	-	**0.935**	< 0.0001	0.878	< 0.0001	0.852	< 0.0001
**QKNG**	**0.935**	< 0.0001	-	-	0.894	< 0.0001	0.868	< 0.0001
**QTSR**	0.878	< 0.0001	0.894	< 0.0001	-	-	**0.938**	< 0.0001
**ETSR**	0.852	< 0.0001	0.868	< 0.0001	**0.938**	< 0.0001	-	-

### Antibody depletion experiments

The next step consisted of a series of antibody depletion experiments, to evaluate the site-shared (cross-reactive) versus allele-specific responses to the four *Pf*MSP1-19KD allele-types. In essence, the antibodies to sites shared between the antigens on the primary and secondary ELISA plates are absorbed (depleted) on the primary plate, so that only antibody to the differing antigen sites are left to bind to the secondary plate.

Eighteen different serum samples were tested, 12 of which had tested high positive when evaluated by ELISA for *Pf*MSP1-19KD IgG to each of the four different allele-types, and the remaining six which tested low positive. These individuals were all infected with the Q-KNG allele-type parasite at some time between one and three months before this sera sample was collected (Figure 2).

**Figure 4:**
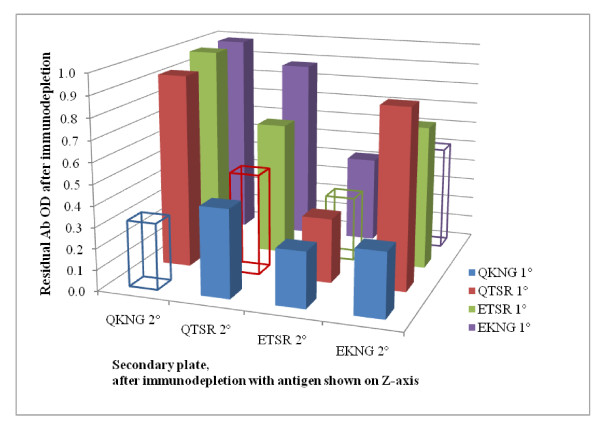
**Immunodepletion results**. The mean residual antibody OD values are shown on the y-axis, antigens that underwent immunodepletion are shown on the z-axis, and secondary antibody responses are shown on the x-axis. The range for all samples was 0.019-0.142. The standard error of most comparisons was low (with a range of 0.00-0.05), and so not shown on this graphic.

Patient sera depleted with the Q-KNG allele-type did not have a positive antibody response remaining to any of the four allele-types, indicating that there was not a -TSR or E- specific antibody response. With respect to the Q- or -KNG specific responses, it was observed that there remained a strong E-KNG response in eight of the 18 individuals after depleting with Q-TSR, and, similarly, after depleting with E-KNG 7 of the 18 individuals had a Q-TSR antibody response. Considering all the allele-specific antibody depletion permutations (as shown in Figure 4), after antibody depletion with Q- and/or -TSR allelic forms, 14 individuals had antibody responses. Of these 14 individuals, 11 had both -KNG and Q- specific responses while all 14 had Q- specific antibody responses (even in the absence of the -KNG). Figure 4 shows the mean antibody level after each of the antibody depletions.

## Discussion

The genetic diversity of *Pf*MSP1-19KD and the potential allele-specific immunity versus shared site immunity must be known for any population undergoing a vaccine trial. Studies in regions of high transmission have suggested that naturally occurring protective antibody responses against the *Pf*MSP1-19KD vaccine candidate might be allele-specific to one or more of the four main allelic forms: E-KNG, E-TSR, Q-KNG, and Q-TSR. The global distribution and evidence for differential recognition of these four major allele-types provides further support for allele-specific immunity and clinical protection [4,7-11,26-31]. Although bearing hallmarks of allele-specific selection, the genetic diversity within *Pf*MSP1-19KD could be explained by genetic drift. Moreover, there has only been one study that investigated this allele-specific immunity in naturally infected individuals. This well-designed study showed evidence for allele-specific and shared site antibody responses, but only three individuals were tested [14]. Additionally, there might be allele-specific antibody responses only at the time of acute infection, with no association to protective immunity upon subsequent infection.

In Iquitos, Peru, after 40 years of effective malaria elimination campaigns, *P. falciparum *reemerged in 1994 and continued as an epidemic until 1998 [15,16]. Since 1998, there has been sustained low transmission of *P. falciparum *(< 0.5 infections/person/year) [16]. It was demonstrated that there is considerable genetic diversity in the polymorphic *Pfmsp1*-B2. Despite this potential for elevated diversity in this region of low transmission, it did not predict an increase in the population-level diversity within *Pfmsp1*-19KD. However, no population-level diversity associated with the *Pfmsp1*-42KD was found, as all 160 samples were of the Mad20 allele-type. An insignificant amount of population-level diversity in the *Pfmsp1*-19KD was found, with the Q-KSNG-L variant comprising 99.4% of the total population-level diversity and the E-KSNG-F variant comprising 0.6% of the population-level diversity. Using samples from this study site and three other study sites, Chenet *et al *[32] reported finding only the Q-KSNG-L allelic form of *Pf*MSP1-19KD in all four study sites.

This extreme conservation is surprising. Even though low genetic diversity in the C-terminal domain was expected, there was enough diversity in the *Pf*MSP1-B2 (all three main families: K1, Mad20 and RO33) for us to expect increased diversity in *Pf*MSP1-19KD, consistent with what may have entered during the epidemic between 1994 and 1998 and/or migrated in (geneflow) during the sustained post-epidemic, low transmission years. This epidemic in the Peruvian Amazon likely included parasites migrating from Brazil and other South American countries [15,16]. However, even if one or more of the other *Pfmsp1*-19KD allele-types were introduced into this population and not maintained due to an immune response selection, it is likely that they would be lost due to genetic drift in low transmission. The diversity in *Pfmsp1*-B2 and *Pfmsp1*-19KD in various studies is shown in Additional File 1.

Previous work has shown a strong association between this population's antibody response to *Pf*MSP1-19KD and protection against febrile illness and parasite density [24]. It has also been shown that anti-*Pf*MSP1-19KD antibody responses appear long-lasting (at least two months in most individuals and longer than four months in many individuals) with more than 50% of the infections detected being asymptomatic [24]. It is possible that these factors might explain the lack of genetic diversity in the *Pfmsp1*-19KD, but regardless if there was protection to allele-specific sites within *Pf*MSP1-19KD, some selection for *Pf*MSP1-19KD allelic diversity would have been anticipated. Of course, it is possible that this protection (asymptomatic infections associated with the antibody response) is not anti-*Pf*MSP1-19KD mediated - and so there would be no immunologic selection on *Pf*MSP1-19KD at all. It is also possible that the antibody response is to shared sites/conserved antigenic determinants and so any pre-existing alternate allele and/or mutation to another allele-type has not been selected for over time and, therefore, lost.

To evaluate whether there are allele-specific responses, the IgG antibody responses to each of the alleles was investigated, and then a series of antibody depletion ELISAs were performed. Although the highest average antibody levels were shown by Q-KNG and Q-TSR, categorical responses to E-KNG and Q-KNG were more similar to each other than to Q-TSR or to E-TSR. In turn, responses to Q-TSR and E-TSR were found to be more similar than to E-KNG or Q-KNG. Earlier studies concluded that this suggests less cross reactivity between the secondary epidermal growth factor (EGF) domain of the 19KD region of *Pf*MSP1 (-KNG versus -TSR) [13]. However, to truly determine if there is a -KNG versus -TSR or E-K versus Q-K allele-specific response, a more sophisticated antibody depletion experiment is required.

Because Q-KNG is the only *Pf*MSP1-19KD allele detected in this region since 2004, all measured antibody responses must be to shared sites or Q-KNG-specific. Indeed, it was found that after immunodepletion with the recombinant Q-KNG allele there was little response to any of the four allelic-forms of *Pf*MSP1-19KD. In addition, it was observed that anti-Q-KNG antibodies in patient sera were not depleted by either E-KNG or E-TSR, although Q-TSR depleted Q-KNG antibodies in 10 of 18 individuals. Therefore, the majority of the response appears to be to conserved/shared antigenic sites, with some allele specific response, particularly to the Q-. Although a parasite having E-KNG was detected in year 2003, there was no allele-specific response detected to E- in these individuals.

Whether or not this "conserved" antibody response to *Pf*MSP1-19KD is protective is a subject which requires further study. An ongoing study in this population has provided evidence that this response is associated with protection against clinical illness and parasitaemia. For example, children with a positive *Pf*MSP1-19KD antibody response one to two weeks prior to infection (i.e. the child was previously infected with *P. falciparum*) were just as likely to have an asymptomatic infection as an adult in this population. Conversely, adults with a negative antibody response 1-2 weeks prior to infection were just as likely to have a symptomatic infection as a child in this population. This and other findings agree with the many other studies suggesting that antibodies to *Pf*MSP1-19KD are associated with protection [6,5,10,34-36]. A study of the same individuals' successive infections over time is being performed to thoroughly test if antibody responses to shared sites of *Pf*MSP1-19KD are associated with protection. This involves testing how immune responses develop over time to various malaria antigens and how one or more of the antibody responses are associated with the development of exposure-related immunity. Furthermore, this highly Q-KNG exposed population will make it possible to take the anti-*Pf*MSP1-19KD antibody and determine if it will cause growth inhibition of *P. falciparum *parasites during *in vitro *culture.

## Conclusions

A non-allele specific antibody response in *Pf*MSP1-19KD may explain why other allelic forms have not been maintained or evolved in this population. This has important implications for the use of *Pf*MSP1-19KD as a vaccine candidate. It is possible that Peruvians have increased antibody responses to the shared sites of *Pf*MSP1-19KD, either due to exposure/parasite characteristics (like complexity of infection, Branch *et al *[23]) or due to a human-genetic predisposition. Alternatively, these allelic polymorphisms are not immune-specific even in other geographic regions, implying these polymorphisms may be less important in immune evasion that previous studies suggest.

## Competing interests

The authors declare that they have no competing interests.

## Authors' contributions

Study design: OLB, PLS, and EHC. Performed genetic diversity experiments and analysed the data: PLS. Performed immunological assay experiments: EHC and CS. Analysed immunological assay data: EHC and OLB. Wrote the manuscript: PLS, EHC, and OLB. All authors have read and approved the final manuscript.

## Supplementary Material

Additional file 1 **Comparative summary table of PLD studies using *Pf*MSP1-B2 and *Pf*MSP1-19KD **. This data provides a global genetic diversity summary from a selection of studies that have investigated diversity within *Pf*MSP1-B2 and/or *Pf*MSP1-19KD. Click here for file
