# Supplementary material for: The Plasmodium falciparum merozoite surface protein-1 19 KD antibody response in the Peruvian Amazon predominantly targets the non-allele specific, shared sites of this antigen
Source: Malar J. 2010 Jan 4;9:3. doi: 10.1186/1475-2875-9-3 (PMC2818648; doi:10.1186/1475-2875-9-3)
Supplement: Additional file 1 — Comparative summary table of PLD studies using PfMSP1-B2 and PfMSP1-19KD . This data provides a global genetic diversity summary from a selection of studies that have investigated diversity within PfMSP1-B2 and/or PfMSP1-19KD. [file 1475-2875-9-3-S1.DOC]

**Additional files**

Additional file 1

File format: DOC

Title: Comparative summary table of PLD studies using *Pf*MSP1-B2 and *Pf*MSP1_19kD_

Description: This data provides a global genetic diversity summary from a selection of studies that have investigated diversity within *Pf*MSP1-B2 and/or *Pf*MSP1_19kD_.

|  | **Summary of PLD in *Pf*MSP1-B2** | | | | | | | | | | | | | **Summary of PLD in *Pf*MSP1_19kD_** | | |
| --- | --- | --- | --- | --- | --- | --- | --- | --- | --- | --- | --- | --- | --- | --- | --- | --- |
| Region | Study Site | Endemicity/ Reference | K1 | | Mad20 | | | RO33 | | | MRrec | | TOTAL | Study Site | Endemicity/ Reference | No. of haplotypes |
|  |  |  | Dist. | No. K1 alleles | Dist. | No. Mad20 alleles | | Dist. | No. R033 alleles | | Dist. | No. of alleles | No. of alleles |  |  |  |
| East Africa | **Western Kenya** | Hyperendemic | 95.0% | 20 | 72.0% | 15 | | 79.0% | 1 | | 29.0% | 7 | 36 |  |  |  |
|  |  | Takala *et al* 2006 |  |  |  |  |  |  |  |  |  |  |  | **Kenya** | Hyperendemic | 8 |
|  | **Western Kenya** | Hyperendemic | 93.0% | 23 | 79.0% | 20 | | 82.0% | 1 | | N/D | N/D | 44 |  | Qari *et al* 1998 |  |
|  |  | Branch *et al* 2001 |  |  |  |  |  |  |  |  |  |  |  |  |  |  |
|  | **West Uganda** | Mesoendemic | 81.1% | 10 | 41.3% | 8 | | 35.5% | 4 | | N/D | N/D | 22 | **Tanzania** | Hyperendemic | 7 |
|  |  | Peyerl *et al* 2001^1^ |  |  |  |  |  |  |  |  |  |  |  |  | Tanabe *et al* 2007 |  |
| West Africa | **Gabon** | Hyperendemic | 90.4% | 14 | 63.5% | 8 | | 36.5% | 3 | | N/D | N/D | 25 |  |  |  |
|  |  | Aubouy *et al* 2003^2^ |  |  |  |  |  |  |  |  |  |  |  | **Mali** | Hyperendemic | 19^†^ |
|  | **Senegal** | Hyperendemic | 68.0% | 24 | 25.0% | 20 | | 36.0% | 1 | | N/D | N/D | 45 |  | Takala *et al* 2007 |  |
|  |  | Henry *et al* 2006^3^ |  |  |  |  |  |  |  |  |  |  |  | ^†^Used high throughput pyrosequencing and examined 6 SNPs  ^†^Study used high-throughpu | | |
| Central Africa | **Central African Republic** | Hyperendemic  Dolmazon et al 2008^4^ | 14.9% | 5 | 75.0% | 9 | | 38.8% | 3 | | N/D | N/D | 17 |  |  |  |
|  |  |  |  |  |  |  |  |  |  |  |  |  |  |  |  |  |
| Middle East | **Iran** | Mesoendemic  Heidari et al 2007^5^ | 50.0% | 4 | 38.8% | 4 | | 11.2% | 1 | | N/D | N/D | 9 |  |  |  |
|  |  |  |  |  |  |  |  |  |  |  |  |  |  |  |  |  |
| India and Pacific Asia |  |  |  |  |  |  | |  |  | |  |  |  | **Solomon Islands** | Hyperendemic | 4 |
|  |  |  |  |  |  |  | |  |  | |  |  |  |  | Tanabe *et al* 2007 |  |
|  | **India** | Hyperendemic | 17.0% | few^*^ | 50.0% | few^*^ | | 33.0% | few^*^ | | N/D | N/D | 3 to 9 | **Thailand** | Mesoendemic | 3 |
|  |  | Raj *et al* 2004^6^ |  |  |  |  |  |  |  |  |  |  |  |  | Sakihama *et al* 1999 |  |
|  | **India** | Mesoendemic | 17.0% | few^*^ | 49.0% | few^*^ | | 34.0% | few^*^ | | N/D | N/D | 3 to 9 | **Thailand** | Hyperendemic | 5 |
|  |  | Raj *et al* 2004 |  |  |  |  |  |  |  |  |  |  |  |  | Tanabe *et al* 2007 |  |
|  |  | *few = although not given number of alleles exactly, there were < 3 alleles per main family | | | | | | | | | | | | **Vietnam** | Mesoendemic | 4 |
|  |  |  |  |  |  |  |  | | |  |  |  |  |  | Ferreira *et al* 2003 |  |
| Central and South America | **Honduras** | Hypoendemic | 73.2% | 2 | 46.4% | 2 | 0.0% | | | 0 | - | - | 4 |  |  |  |
|  |  | Haddad *et al* 1999^7^ |  |  |  |  |  |  |  |  |  |  |  |  |  |  |
|  | **Columbia** | Hyperendemic | 11.0% | 1 | 63.0% | 2 | 26.0% | | | 1 | N/D | N/D | 4 |  |  |  |
|  |  | Gomez *et al* 2002^8^ |  |  |  |  |  |  |  |  |  |  |  |  |  |  |
|  | **Venezuelan Amazon** | Mesoendemic | 83.3% | 2 | 7.1% | 3 | 4.8% | | | 1 | N/D | N/D | 6 |  |  |  |
|  |  | Tami *et al* 2002^9^ |  |  |  |  |  |  |  |  |  |  |  |  |  |  |
|  | **Brazilian Amazon** | Hypoendemic | 48.1% | N/D | 19.5% | N/D | 32.5% | | | N/D | N/D | N/D | at least 3 | **Brazil** | Hypoendemic | 7 |
|  |  | Ferreira *et al* 1998^10^ |  |  |  |  |  |  |  |  |  |  |  |  | Silveira *et al* 2001 |  |
|  | **Peruvian Amazon** | Hypoendemic | 63.0% | 4 | 37.0% | 2 | 0.0% | | | 0 | N/D | N/D | 6 | **Peruvian Amazon** | Hypoendemic | 1 |
|  |  | Chenet *et al* 2008 |  |  |  |  |  |  |  |  |  |  |  |  | Chenet *et al* 2008 |  |
|  | **Peruvian Amazon** | Hypoendemic | 52.5% | 3 | 45.3% | 3 | 2.2% | | | 1 | 0.0% | 0 | 7 | **Peruvian Amazon** | Hypoendemic | 2 |
|  |  | **Current Study** |  |  |  |  |  |  |  |  |  |  |  |  | **Current Study** |  |

**^1^**Peyerl-Hoffmann G, Jelinek T, Kilian A, Kabagambe G, Metzger WG, von Sonnenburg F**:** Genetic diversity of *Plasmodium falciparum* and its relationship to parasite density in an area with different malaria endemicities in West Uganda. *Trop Med Int Health* 2001**,** 6:607-**6**13.

# ^2^Aubouy A, Migot-Nabias F, Deloron P: Polymorphism in two merozoite surface proteins of *Plasmodium falciparum* isolates from Gabon. *Malar J* 2003, 2:12.

# ^3^Henry M, Diallo I, Bordes J, Ka S, Pradines B, Diatta B, M'Baye PS, Sane M, Thiam M, Gueye PM, Wade B, Touze JE, Debonne JM, Rogier C, Fusai T: Urban malaria in Dakar, Senegal: chemosusceptibility and genetic diversity of *Plasmodium falciparum* isolates. *Am J Trop Med Hyg* 2006, 75:146-151.

# ^4^Dolmazon V, Matsika-Claquin MD, Manirakiza A, Yapou F, Nambot M, Menard D: Genetic diversity and genotype multiplicity of *Plasmodium falciparum* infections in symptomatic individuals living in Bangui (CAR). *Acta Trop* 2008, 107:37-42.

# ^5^Heidari A, Keshavarz H, Rokni MB, Jelinek T: Genetic diversity in merozoite surface protein (MSP)-1 and MSP-2 genes of *Plasmodium falciparum* in a major endemic region of Iran. *Korean J Parasitol* 2007, 45:59-63.

# ^6^Raj DK, Das BR, Dash AP, Supakar PC: Genetic diversity in the merozoite surface protein 1 gene of *Plasmodium falciparum* in different malaria-endemic localities. *Am J Trop Med Hyg* 2004, 71:285-289.

# ^7^Haddad D, Snounou G, Mattei D, Enamorado IG, Figueroa J, Ståhl S, Berzins K: Limited genetic diversity of *Plasmodium falciparum* in field isolates from Honduras. *Am J Trop Med Hyg* 1999, 60:30-34.

# ^8^Gómez D, Chaparro J, Rubiano C, Rojas MO, Wasserman M: Genetic diversity of *Plasmodium falciparum* field samples from an isolated Colombian village. *Am J Trop Med Hyg* 2002, 67:611-616.

# ^9^Tami A, Grundmann H, Sutherland C, McBride JS, Cavanagh DR, Campos E, Snounou G, Barnabé C, Tibayrenc M, Warhurst DC: Restricted genetic and antigenic diversity of *Plasmodium falciparum* under mesoendemic transmission in the Venezuelan Amazon. *Parasitology* 2002, 124:569-581.

# ^10^Ferreira MU, Liu Q, Kaneko O, Kimura M, Tanabe K, Kimura EA, Katzin AM, Isomura S, Kawamoto F: Allelic diversity at the merozoite surface protein-1 locus of *Plasmodium falciparum* in clinical isolates from the southwestern Brazilian Amazon. *Am J Trop Med Hyg* 1998, 59:474-48
